# Clinical features of headache associated with mobile phone use: a cross-sectional study in university students

**DOI:** 10.1186/1471-2377-11-115

**Published:** 2011-09-26

**Authors:** Min Kyung Chu, Hoon Geun Song, Chulho Kim, Byung Chul Lee

**Affiliations:** 1 Department of Neurology, Hallym University College of Medicine, Anyang, Korea; 2 Department of electronic, electrical, control & instrumentation engineering, Hanyang University, Ansan, Korea

## Abstract

**Background:**

Headache has been reported to be associated with mobile phone (MP) use in some individuals. The causal relationship between headache associated with MP use (HAMP) and MP use is currently undetermined. Identifying the clinical features of HAMP may help in clarifying the pathophysiology of HAMP and in managing symptoms of individuals with HAMP. The aim of the present study is to describe the clinical features of HAMP.

**Methods:**

A 14-item questionnaire investigating MP use and headache was administered to 247 medical students at Hallym University, Korea. Individual telephone interviews were subsequently conducted with those participants who reported HAMP more than 10 times during the last 1 year on the clinical features of HAMP. We defined HAMP as a headache attack during MP use or within 1 hour after MP use.

**Results:**

In total, 214 (86.6%) students completed and returned the questionnaire. Forty (18.9%) students experienced HAMP more than 10 times during the last 1 year in the questionnaire survey. In subsequent telephone interviews, 37 (97.4%) interviewed participants reported that HAMP was triggered by prolonged MP use. HAMP was usually dull or pressing in quality (30 of 38, 79.0%), localised ipsilateral to the side of MP use (32 of 38, 84.2%), and associated with a burning sensation (24 of 38, 63.2%).

**Conclusion:**

We found that HAMP usually showed stereotyped clinical features including mild intensity, a dull or pressing quality, localisation ipsilateral to the side of MP use, provocation by prolonged MP use and often accompanied by a burning sensation.

## Background

The use of mobile phones (MPs) has increased dramatically, with over 3 billion people currently using MPs worldwide [1]. In Korea, 92.9% of the population of approximately 48.4 million people used MPs in 2008 [2]. With the increased use of MPs, some users have reported the development of symptoms such as headache, sleep disturbance, memory loss, dizziness, and burning sensations during or after MP use [3,4]. Several epidemiological studies have suggested that MP use may be related to the occurrence of these symptoms [5].

Experiments aimed at explaining these symptoms have shown changes in cerebral blood flow [6,7], altered electroencephalogram (EEG) patterns [3,5,6], and changes in responses to transcranial magnetic stimulation [8] resulting from MP use. A series of double-blind, sham-controlled studies have reported no significant differences in headache provocation between control and MP radiofrequency fields (RFs) among participants who experienced some of the aforementioned symptoms during MP use [9-15]. Although these studies assessed provocation of headache by RFs similar to those generated by MPs, the relationship between headache provocation and real MP use is currently undetermined [16,17]. Recent proposals for standardised general diagnostic criteria for the secondary headaches included typical headache characteristics of the presumed causative factors in addition to the temporal relationship of headache and causative factors in onset, worsening and improvement of headache [18]. Identifying the clinical features of headache associated with mobile phone use (HAMP) will help in understanding the pathophysiology of HAMP and in managing symptoms of individuals with HAMP in addition to clarifying the relationship between headache provocation and MP use.

## Methods

**Figure 1:**
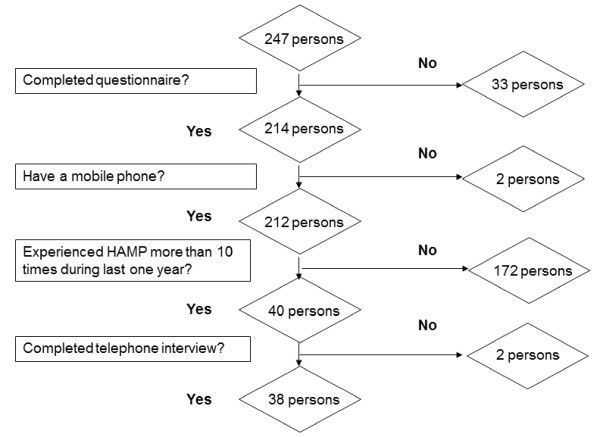
**Study flow diagram**.

This study was divided into 2 parts: an initial questionnaire survey and subsequent telephone interviews of participants who reported that they experienced HAMP more than 10 times during the last 1 year. HAMP was defined as any headache during or within 1 hour after MP use. In the initial questionnaire survey, participants were asked about their MP use, current headaches not associated with MP use (HNAMP) and HAMP. If a participant reported HAMP more than 10 times during the last 1 year, he or she was followed up by telephone interview within 2 weeks of the initial questionnaire survey. The telephone interview was designed for a detailed identification of HAMP (Figure 1). The study was conducted in accordance with the guidelines of the Institutional Review Board of Hallym University Sacred Heart Hospital and with the principles in the Declaration of Helsinki [19]. The authors had full access to all the data in the study and take responsibility for the integrity of the data and the accuracy of the data analysis. This study was conducted in September 2005.

### Subjects

We investigated the first-, second-, and third-year medical students at Hallym University College of Medicine in Chuncheon, Korea. At the time of the initial questionnaire survey, we asked all participants for written signed consent for participation and excluded those students who: (1) declined to participate, (2) did not use MPs, or (3) did not provide written consent for participation. An investigator (CK) who was not involved in medical student education at that time recruited participants and collected signed consent forms.

### Questionnaire survey

A 14-item questionnaire was administered to all participants. The questionnaire included 9 items concerning characteristics of HNAMP such as frequency, duration, quality, location and severity of headache; nausea; vomiting; photophobia; and phonophobia (Additional file 1). The questionnaire also included 3 items on the current status of MP use (average daily duration, frequency of MP use and overall duration of MP use) and collected data on the participants' gender and age. An individual was defined as having HAMP if he or she experienced HAMP more than 10 times during the last 1 year.

### Telephone interview

A neurologist (MKC) conducted semi-structured telephone interviews with respondents who reported that they currently or previously experienced HAMP in the questionnaire survey. The telephone interview included 18 questions on clinical features (onset time, frequency, severity, quality, and location of headache), triggering factors (prolonged MP use, frequent MP use, and other triggering factors), and associated symptoms (burning sensation, dizziness, nausea, vomiting, photophobia, phonophobia, osmophobia and orbital or periorbital pain) of HAMP (Additional file 2).

### Statistical analysis

Categorical variables were summarised by the corresponding percentages, and continuous variables were generally summarised by descriptive statistics (mean and standard deviation [SD]). Between-group differences were evaluated using analysis of variance (ANOVA) after confirming that the data were normally distributed. R version 2.9.1 and R Commander version 1.4-10 statistical software for Windows (GNU general public license) were used for all analyses. The significance of each factor was analysed using 2-tailed p values, where p < 0.05 was considered statistically significant.

## Results

### Demographic features

A total of 247 medical students at Hallym University College of Medicine were recruited, and 214 students agreed to participate in the study. Two participants did not use a MP at the time of the survey and were thus excluded from the study. All of the remaining 212 participants completed the questionnaire. Their mean age was 23.6 ± 2.6 years; 84 participants (39.6%) were women and 128 (60.4%) were men. In the questionnaire survey, 40 participants indicated that they experienced HAMP more than 10 times during the last 1 year. Of these, 38 participants completed the telephone interview (Figure 1).

### Mobile phone use

In the 212 participants, the mean daily duration of MP use was 33.4 ± 34.5 minutes, and the mean daily frequency of MP use was 7.4 ± 6.3 calls. The mean duration of MP use was 5.5 ± 4.0 years. No significant differences in age, gender, daily duration of MP use, daily frequency of MP use, or overall duration of MP use were observed between participants who experienced HAMP and those who did not (Table 1).

**Table 1:** Duration of daily mobile phone use, frequency of daily mobile phone use, and duration of mobile phone use according to the HAMP status in questionnaire survey.

	HAMP participants (n = 40)	Non-HAMP participants (n = 172)	p value
Age	23.88 ± 2.59	23.42 ± 3.14	NS
Female, N (%)	16 (40.0%)	105 (39.0%)	NS
Duration of daily mobile phone use (minutes per day)	31.2 ± 31.4	34.0 ± 35.3	NS
Frequency of daily mobile phone use (number per day)	7.1 ± 5.7	7.4 ± 6.4	NS
Duration of mobile phone use (years)	6.6 ± 6.9	5.3 ± 2.8	NS

### Headache not associated with mobile phone use (HNAMP)

Among the 212 MP users who completed the questionnaire, 191 answered that they had experienced headache in the previous year, and 135 reported that their HNAMP lasted more than 30 minutes. Three participants with HAMP reported that they did not experience HNAMP and only suffered HAMP during the previous year. We observed no significant differences between participants who experienced HAMP and those who did not when analysing the following: prevalence of headaches lasting more than 30 minutes during the last 1 year, moderate or severe intensity of headache, pulsating quality of headache, aggravation of headache by routine physical activity, unilateral pain, nausea, and photophobia. Phonophobia was more prevalent among participants with HAMP compared to participants without HAMP. The proportion of participants identified in the questionnaire survey as experiencing migraine according to the ID-Migraine™ criteria [20] (experiencing at least 2 of the following conditions during headache: nausea, photophobia, and functional impairment) was not significantly different between these 2 groups (Table 2).

**Table 2:** Clinical features of headache not associated with mobile phone use (HNAMP).

	HAMP participants N (%)	Non-HAMP participants N (%)	p value
Headache not associated with mobile phone use, lasing ≥ 30 min.	23 (57.5)*	112 (65.1)*	NS
Moderate or severe intensity	13 (35.1)^†^	51 (33.1)^†^	NS
Unilateral pain	19 (51.4)^†^	68 (44.2)^†^	NS
Pulsating quality	14 (37.8)^†^	52 (33.8)^†^	NS
Aggravation of headache by routine activity	9 (24.3)^†^	24 (15.64)^†^	NS
Nausea	4 (10.8)^†^	12 (7.8)^†^	NS
Photophobia	6 (16.2)^†^	24 (15.6)^†^	NS
Phonophobia	28 (75.7)^†^	72 (46.8)^†^	0.002
ID-migraine (%)	4 (10.8)^†^	13 (8.4)^†^	NS

NS: not significant. *From all participants at initial questionnaire survey (40 participants with HAMP and 172 participants without HAMP). ^†^From participants with headache not associated with mobile phone use (37 HAMP participants and 154 Non-HAMP participants).

### Headache associated with mobile phone use (HAMP)

#### (1) Headache features of HAMP

Among the 212 MP users, 40 (18.9%) participants reported in the questionnaire survey that they experienced HAMP more than 10 times during the last 1 year. Of the 40 participants with HAMP, 38 completed the telephone follow-up interview.

**Figure 2:**
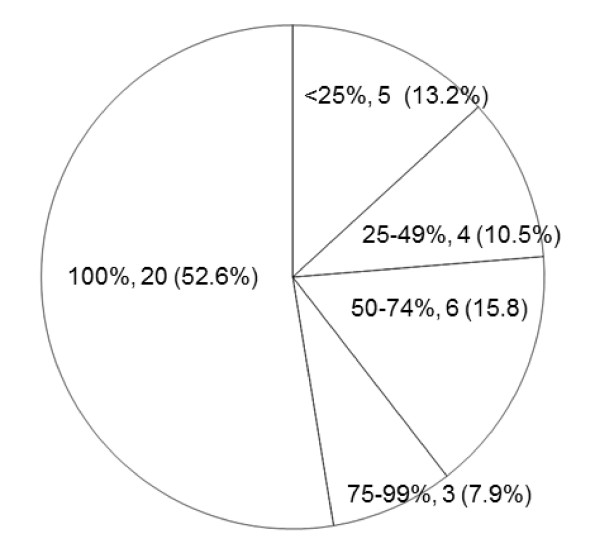
**Frequency of HAMP during or after MP use**.

**Figure 3:**
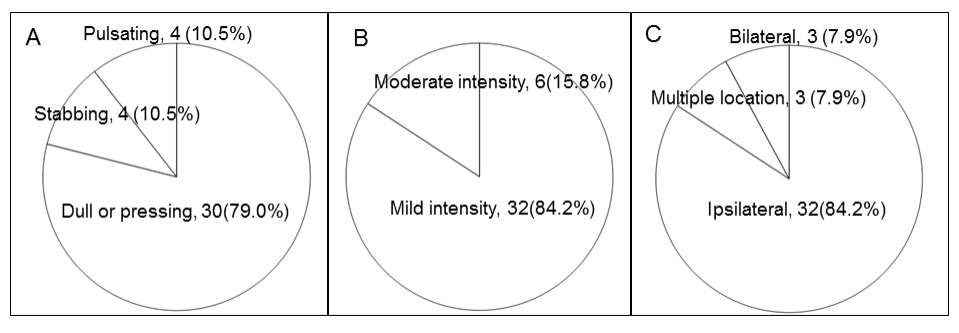
**Quality (A), intensity (B) and location (C) of HAMP**.

A telephone interview was conducted to determine the time of onset, frequency, location, characteristics, associated symptoms, and triggering factors of HAMP. Eighteen (47.4%) participants reported that their HAMP developed during MP use, 11 (28.9%) reported that HAMP developed after MP use, and 9 (23.7%) reported that HAMP developed either during or after MP use. Twenty (52.6%) participants reported that HAMP occurred every time they used a MP (Figure 2). The majority of participants reported that HAMP was dull or pressing in quality, mild in intensity and localised to the side of MP use (Figure 3-A, B, C). Four participants reported aggravation of headache by physical activity.

#### (2) Associated symptoms of HAMP

During the telephone interview, 27 (71.1%) participants with HAMP reported experiencing a burning sensation every time they used a MP. In conjunction with HAMP, 15 (39.5%) participants reported dizziness and 12 (31.6%) reported orbital or periorbital pain (Table 3).

**Table 3:** Associated symptoms of HAMP in telephone interview.

Associated symptoms	No. of participants (%)
Burning sensation	27 (71.1)
Dizziness	15 (39.5)
Orbital or periorbital pain	12 (31.6)
Phonophobia	9 (23.7)
Nausea	4 (10.5)
Photophobia	1 (2.6)
Osmophobia	0 (0.0)

#### (3) Triggers of HAMP

**Figure 4:**
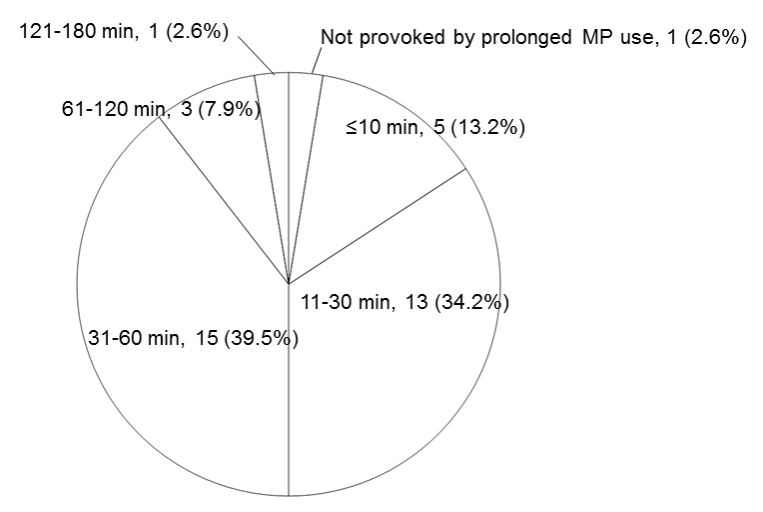
**Time to onset of HAMP after starting MP use**.

Thirty-seven (97.4%) telephone-interviewed participants claimed that prolonged MP use triggered HAMP. Three (7.9%) participants reported that frequent use of MP also triggered HAMP. The mean period of MP use necessary to trigger HAMP was 49.7 ± 36.7 minutes, and the median time was 40 minutes (Figure 4). One participant with HAMP reported that headache also developed when using a regular telephone. When asked whether the use of hands-free equipment during MP calls affected HAMP, 16 participants reported that headache did not develop when they used hands-free equipments, and the remaining 22 participants had never used hands-free equipments.

## Discussion

The present study demonstrated that 18.9% of the participants in the initial questionnaire survey experienced HAMP more than 10 times during the last 1 year. HAMP exhibited mostly stereotyped clinical features such as mild intensity, localisation ipsilateral to the side of MP use, dull or pressing quality, association with a burning sensation, and provocation by prolonged MP use.

These results are similar to those of a study on MP users in Sweden and Norway, in which 8.4-13% of MP users reported HAMP [21]. In Saudi Arabia, HAMP was observed in 22.4% of MP users [22]. Santini et al. reported that 10-20% of MP users complained of HAMP in a questionnaire study conducted in France [23]. Discrepancies in the proportion of MP users who experience HAMP may be due to differences in MP types, demographic features of users, social level of concerns about MP use or media reporting about them [24]. In addition to headache, a burning sensation and dizziness have been commonly observed among MP users. Burning sensation and dizziness accompanying headache were also frequently observed in our study.

Headache during or after MP use could be induced by altering conditions during MP use including radiofrequency fields (RFs), psychological factor, temperature change, noise and various combinations thereof.

Exposure to RFs during MP use has been suggested to trigger a variety of symptoms such as headache, fatigue, concentration difficulties, and nausea [11]. A series of double-blind provocation studies failed to demonstrate a causal relationship between RFs exposure and these symptoms [9,10,12-15]. A meta-analysis of 46 blind or double-blind provocation studies reported no robust evidence in support of a connection between RFs exposure and symptoms as a biological entity [17].

To date, no specific effects of active RFs exposure have been identified, nor has any difference in triggering symptoms been noted between active and sham conditions. Instead, various responses have been observed in repeated exposures of the same participants. Thus, these provocation studies suggest the role of psychological factor, a nocebo effect, in symptom provocation [25-27]. Nocebo effect refers to harmful, unpleasant, or undesirable effects a subject manifests after receiving a sham treatment [26,28]. In the present study, we used an initial questionnaire survey and subsequent telephone interviews, and did not evaluate psychological factors or nocebo effects associated with HAMP provocation.

Additional altering conditions on subjects during MP use are local temperature change and noise [29], both of which can provoke headache [30-33]. Local temperature elevation in the area of MP use has been noted in several studies [34,35]. The sound of a MP is not the same as an actual human voice and usually includes noise [36]. One double-blind cross-over provocation study has taken into account thermal effects of MP use in a provocation test [11]. These investigators used a heated (39 ± 0.2°C) ceramic plate to mimic the sensation caused by using a warm MP during active and sham exposures of RFs. However, they did not investigate whether temperature change during MP use induced symptoms solely or in combination with RFs exposure. The effects of local temperature change and noise in connection with MP use in symptoms triggering by blinded tests were not reported yet. In our study, a burning sensation was reported by 71.1% of participants with HAMP and phonophobia was more prevalent among participants with HAMP compared to those without HAMP. These findings suggest the possibility that local temperature change and/or noise resulting from MP use are associated with HAMP provocation.

Proposals for new standard general diagnostic criteria for secondary headaches include suggestions in evidence of causation of headache: (1) headache has occurred in temporal relationship to the onset of the presumed causative disorder, (2) headache has occurred or has significantly worsening of the presumed causative factors, (3) headache has improved in temporal relation to improvement of the presumed causative factors, (4) headache has characteristics of typical of the causative disorder, (5) other evidence of causation. Evidence of causation of headache is inferred when at least 2 of the aforementioned 5 conditions are met [18]. Regarding the results of our study, HAMP satisfied: (1) temporal relationship to onset of headache (experiencing HAMP more than 10 times during or after MP use), (2) occurring or worsening by worsening of the presumed factor (more individuals experienced HAMP with longer duration of MP use), and (3) characteristics typical of the causative disorder (stereotyped headache features and associated symptoms).

Headache provocation by regular telephone use was reported by only 1 participant with HAMP and HAMP did not occur when using hands-free equipment in the present study. Rare headache provocation by regular telephone use suggests that HAMP is related to certain conditions that are specific to MP use and not to regular telephone use. The lack of HAMP occurrence when using hands-free equipments also suggests that certain factor(s) nullified by hands-free equipments are related to HAMP provocation.

One possible mechanism for headache provocation by regular telephone use was her chronic headache disorder. Her HNAMP occurred 4 days per week with migrainous features (nausea, aggravation by routine physical activity and phonophobia). Clinical features of her HNAMP suggested that she had chronic migraine [37,38]. Her headache provocation by regular telephone use probably resulted from enhanced sensitivity to environmental stimuli in chronic migraine. The other possible mechanism for her headache provocation by regular telephone use is idiopathic environmental hypersensitivity to electromagnetic fields (IEI-EMF) [17]. However, we did not assess the symptoms during or after using electrical devices other than MP and regular telephone and could not know whether she had IEI-EMF.

Our study has some limitations. First, this study was not a blinded attempt to provoke headache under specific conditions. Instead, we have described the self-reported prevalence and clinical features of HAMP among MP users under actual conditions. Thus, we were not able to evaluate the causal relationship between headache provocation and specific factors related to MP use. Second, the participants in this study were all medical students in Korea. Most of them were 20-30 years of age and used MPs; therefore, our study participants did not reflect the general population. Third, the initial questionnaire survey assessed only the occurrence of headache and HAMP, and we conducted follow-up interviews with only those participants who reported that they were currently experiencing or had previously experienced HAMP. We assessed other MP-associated symptoms such as burning sensation, dizziness, and orbital or periorbital pain during the telephone interviews, but because they were not included in the initial questionnaire survey, we were unable to independently evaluate MP-associated symptoms other than headache among MP users. Fourth, the study was conducted in 2005 when only code division multiple access (CDMA) type MPs were available in Korea. CDMA-type MPs and global system for mobile communication (GSM) type MPs differ in their operating frequency range and electromagnetic filed output power. Thus, the effects of CDMA-type MPs on humans may differ from those of GSM-type MPs. Nevertheless, the prevalence of HAMP among CDMA-type MP users in our study was similar to those of previous reports of HAMP among GSM-type MP users. Fifth, we screened for HAMP by using an initial questionnaire survey in which the participants were asked to recall HAMP during the last 1 year, and there is the possibility of erroneous memory of their headaches. In the present study, most participants with HAMP reported their HAMP occurred frequently during or after MP use (Figure 2). These findings suggest that most participants with HAMP had experienced HAMP recently and might have answered positively in the initial questionnaire survey.

## Conclusions

We conclude that HAMP usually presented with stereotyped clinical features such as mild intensity, a dull or pressing quality, localisation ipsilateral to the side of MP use, provocation by prolonged MP use and accompaniment by a burning sensation. Although earlier double-blind RFs provocation tests failed to demonstrate a relationship between HAMP or MP-associated symptoms and RFs exposure, further randomised, controlled provocation studies that examine the effects of altering conditions by MP use including noise, local temperature change, RFs exposure and combinations thereof, are needed in order to better understand the development and nature of HAMP.

## List of abbreviations

MP: mobile phone; RFs: radiofrequency fields; GSM: global system for mobile communication; CDMA: code division multiple access; HAMP: headache associated with mobile phone use; HNAMP: headache not associated with mobile phone use

## Competing interests

The authors declare that they have no competing interests.

## Authors' contributions

MKC was responsible for the conception of study, design, data analysis and the wring of the manuscript. HGS was responsible for technical consultation. He also made contributions in the conception of study, design and writing the manuscript. CK was responsible for data collection. He also made contribution in the wring the manuscript. BCL was responsible for the conduction of this study. He also made contribution in conception of study, design and the writing the manuscript. All authors read and approved the final manuscript.

## Pre-publication history

The pre-publication history for this paper can be accessed here:

http://www.biomedcentral.com/1471-2377/11/115/prepub

## Supplementary Material

Additional file 1 **Questionnaire for initial screening survey**. Korean version was used in present study and English version is presented for convenience. Click here for file

Additional file 2 **Questionnaire for subsequent telephone interview**. Korean version was used in present study and English version is presented for convenience. Click here for file
