# Supplementary material for: Clinical features of headache associated with mobile phone use: a cross-sectional study in university students
Source: BMC Neurol. 2011 Sep 26;11:115. doi: 10.1186/1471-2377-11-115 (PMC3193165; doi:10.1186/1471-2377-11-115)
Supplement: Additional file 1 — Questionnaire for initial screening survey. Korean version was used in present study and English version is presented for convenience. [file 1471-2377-11-115-S1.DOC]

**Questionnaire for initial screening survey: Korean version**

아래 설문들은 여러분들의 **휴대폰 사용과 휴대폰과 관련 없는 두통**에 대한 설문입니다. 질문에 적절히 답변해주시기 바랍니다.

성명: 생년월일: 년 월

전화 : ( ) -

휴대폰: (010)(011)(016)(017)(018)(019)- -

1. 지난 1년간 **휴대폰사용과 관계없이 두통을** 겪은 적이 있은 적이 있습니까?

(예) (아니오)

2. 지난 1년간 휴대폰사용 중이나 사용 1시간 이내에 10회 이상 두통이 나타났습니까? (예) (아니오)

3. 하루에 평균 휴대폰은 몇 번 정도 사용합니까? 회

4. 하루 평균 휴대폰은 얼마나 사용하십니까? 시간 분

5. 휴대폰 사용하기 시작한 것은 언제부터 입니까?

(년) (월) (주) (월) (주) (일)전부터

다음은 **휴대폰사용과 관계 없는 두통에** 관한 질문입니다. 여러분의 휴대폰 사용과 관계없는 평소의 두통에 대해 적절히 답변해주십시오(7~15번)

6. 평소 두통의 빈도는 대략 어느 정도 입니까?

매일( 회), 매주 ( 회), 매월 ( 회), 매년 ( 회)

(한가지에 선택에서 답해주십시오, 예) 매월 3회

7. 평소의 두통의 기간은 대략 어느 정도 입니까?

( )초, ( )분, ( )시간, ( )일, ( )주, ( )달

한가지에 선택에서 답해주십시오, 평균 시간을 표시해주시기 바랍니다. 예) 3시간

8. 두통이 발생되는 부분은 어디입니까?

1. 머리 전체 (2) 여기저기 (3) 좌측 (4) 우측 (5) 좌든 우든 어쨌든 한쪽만

9. 두통의 양상은 어떻습니까? (현재의 두통의 해당 사항에 전부다 기입하세요)

⬜ 욱신거린다. 맥박에 따라 두근거린다

⬜ 묵직하고 뻐근하다

⬜ 띠처럼 조이는 듯 하다

⬜ 바늘로 찌르는 듯이 쿡쿡 쑤신다

⬜ 갑자기 망치로 내려치듯이 심한 강도로 갑자기 시작하였다

⬜ 스멀거린다, 뭔가가 기어가는듯하다

⬜ 기타

(기타면 두통의 양상을 간단히 적어주세요)

10. 두통은 얼마나 심합니까?

(1) 두통이 있기는 하지만 일상 활동에 지장을 주지는 않는다 (경도).

(2) 두통으로 일상활동에 지장이 있기는 하지만 할 수 있다 (중등도).

(3) 두통으로 인하여 일상 활동을 할 수 없다 (심도).

11. 두통이 계단을 걷는 것과 같은 일상적인 활동에 의해 악화됩니까?

(예) (아니오)

12. 두통이 있을 때 구역질이 나거나 구토가 동반됩니까?

(예) (아니오)

13. 두통이 있을 때 시끄러운 곳에 있으면 더 고통스럽습니까?

(예) (아니오)

14. 두통이 있을 때 밝은 곳에 있으면 더 고통스럽다.

(예) (아니오)

**Questionnaire for initial screening survey: English version.**

These questions are about your **mobile phone use and headache not associated with mobile phone use.** Please answer the question about your mobile phone use and headache not associated with mobile phone use. (No.1~No.5)

Name: Date of birth: (yyyy)/(mm)/(dd) ____/__/__

Gender: (Men) (Women)

Phone number: ( ) -

Mobile phone number: ( ) -

1. Have you had headaches **(not associated with mobile phone use)** during the last 1 year?

(Yes)/ (No)

2. Have you had a headache during mobile phone use or within 1 hour of mobile phone use more than 10 times during the last 1 year?

(Yes)/ (No)

3. What is the average number of times you use a mobile phone in a day? ______ times

4. For how long do you use a mobile phone in a day on an average?

___ hr _ min

5. When did you start to use a mobile phone?

Since (yyyy)/(mm)/(dd) ____/__/__

These questions are about **headaches not associated with mobile phone use.** Please answer the question about **headaches not associated with mobile phone use** (No.6~No.14)

6. How often did you experience such headaches during the last 1 year?

daily( times), weekly ( time), monthly ( times),

yearly ( times)

*Please answer only once for each question, e.g.) monthly 3 times

7. How long did these headaches last on an average?

( ) second(s), ( ) minute(s), ( ) hour(s), ( ) day(s),

( ) week(s), ( ) month(s)

* Please answer only once for each questions. e.g) 3 hours

8. What was the location of the headache?

1. Bilateral, across the head (2) Here and there (migrating)

(3) Left side (4) Right side

(5) Unilateral either way

9. What was the headache not associated with mobile phone use like? (Please statement the most accurately describes your headache not associated with mobile phone use)

⬜ Pulsating and throbbing

⬜ Heavy and stiff

⬜ Tightening feeling like tying a band around your head

⬜ Sharp like pinpricking

⬜ Sudden and severe like hitting your head with a hammer

⬜ Creepy or crawling sensation

⬜ Other description

(If you answer “Other description” to questions, please describe the nature of your headache succinctly)

10. How bad was your headache?

(1) Headache did not disturb usual daily activities (mild).

(2) Headache often disturbed usual daily activities, but I could perform more than half of my daily activities (moderate).

(3) I can’t perform my usual daily activities when I suffer these headaches (severe).

11 The headache worsened by activities such as walking or climbing stairs?

(Yes) (No)

12. Did you feel nauseated or sick to your stomach during your headache?

(Yes) (No)

13. Was your headache more painful when you were in noisy surroundings?

(Yes) (No)

14. Did light bother you a lot more than when you don’t have headaches?

(Yes) (No)
