# Supplementary material for: Clinical features of headache associated with mobile phone use: a cross-sectional study in university students
Source: BMC Neurol. 2011 Sep 26;11:115. doi: 10.1186/1471-2377-11-115 (PMC3193165; doi:10.1186/1471-2377-11-115)
Supplement: Additional file 2 — Questionnaire for subsequent telephone interview. Korean version was used in present study and English version is presented for convenience. [file 1471-2377-11-115-S2.DOC]

**Questionnaire for subsequent telephone interview: Korean version**

다음 질문은 **휴대폰사용과 관련된 두통**에 대한 질문입니다. 여러분의 **휴대폰 사용과 관련 있는 두통**에 대해 적절히 답변해주십시오. (1~18번)

1. 휴대폰사용과 관련된 두통이 나타나기 시작한 것은 언제부터 입니까?

(년) (월) (월) (주)전부터

2. 휴대폰사용과 관련된 두통은 언제 나타납니까? 맞는 부분에 **모두** 답해주십시오.

(1) 휴대폰 사용을 **오래할** 때(한통화가 길어질 때) (예) (아니오)

(2) **자주** 휴대폰을 사용할 때(짧게라도 자주 할 때) (예) (아니오)

3. 만약 휴대폰을 **오래 사용할 때** 두통이 난다면 얼마나 오래 통화하면 나타납니까?

(시간)(분) (예, 1시간)

4. 만약 휴대폰을 **자주 사용할 때** 두통이 난다면 얼마나 자주 통화하면 나타납니까?

(하루당)(1시간당) 회 (예, 1시간당 1회)

5. 휴대폰사용과 관련된 두통이 있다면 언제 두통이 나타납니까?

휴대폰 **사용 중에** 나타난다. (예) (아니오)

휴대폰 **사용 후에** 나타난다. (예) (아니오)

6. 휴대폰사용과 관련된 두통은 얼마나 자주 나타난다면 얼마나 자주 나타납니까?

전체 휴대폰 사용시의 (1/10이하)(1/4 이하) (1/4-1/2) (1/2-3/4) (3/4이상)(항상)

7. 휴대폰사용과 관련된 두통이 발생되는 부분은 어디입니까?

(1) 휴대폰을 귀에 대는 쪽 머리 (2) 휴대폰을 귀에 대는 반대쪽 머리

(3) 머리 전체 (4) 여기저기 (5) 좌든 우든 어쨌든 한쪽만

(6) 일정하지 않다

8. 휴대폰사용과 관련된 두통은 어떠한지요? (휴대폰사용과 관련된 두통에서 주로 나타나는 양상 한가지만 기입하세요)

⬜ 욱신거린다. 맥박에 따라 두근거린다

⬜ 묵직하고 뻐근하다

⬜ 띠처럼 조이는 듯 하다

⬜ 바늘로 찌르는 듯이 쿡쿡 쑤신다

⬜ 갑자기 망치로 내려치듯이 심한 강도로 갑자기 시작하였다

⬜ 스멀거린다, 뭔가가 기어가는듯하다

⬜ 기타

(기타면 두통의 양상을 간단히 적어주세요)

9. 화끈거리는 증상이 휴대폰사용과 관련된 두통 동안 또는 두통 1시간 이후에 있습니까? (예) (아니오)

(만약 화끈거리는 증상이 있다면 10번 문항에 답하십시오. 만약 화끈거리는 증상이 없다면 10번 문항은 답하지 마십시오.)

10. 화끈거리는 증상은 얼마나 자주 있습니까?

전체 휴대폰 사용시의

(1/10이하)(1/4 이하) (1/4-1/2) (1/2-3/4) (3/4이상)(항상)

전체 휴대폰 관련 두통이 나타날 때의

(1/10이하)(1/4 이하) (1/4-1/2) (1/2-3/4) (3/4이상)(항상)

11. 휴대폰사용과 관련된 두통이 나타날 때 어지럼증이 나타납니까?

(예) (아니오)

12. 휴대폰사용과 관련된 두통은 얼마나 심합니까?

(1) 두통이 있기는 하지만 일상 활동에 지장을 주지는 않는다 (경도).

(2) 두통으로 일상활동에 지장이 있기는 하지만 할 수 있다 (중등도).

(3) 두통으로 인하여 일상 활동을 할 수 없다 (심도).

13. 휴대폰사용과 관련된 두통이 계단을 걷는 것과 같은 일상적인 활동에 의해 악화됩니까? (예) (아니오)

14. 휴대폰사용과 관련된 두통이 있을 때 구역질이 나거나 구토가 동반됩니까

(예) (아니오)

15. 휴대폰사용과 관련된 두통이 있을 때 시끄러운 곳에 있으면 더 고통스럽습니까? (예) (아니오)

16. 휴대폰사용과 관련된 두통이 있을 때 밝은 곳에 있으면 더 고통스럽습니까?

(예) (아니오)

17. 휴대폰사용과 관련된 두통이 있을 때 냄새가 다르게 느껴집니까?

(예) (아니오)

18. 휴대폰사용과 관련된 두통이 있을 때 눈 또는 눈 주위가 같이 아픕니까?

(예) (아니오)

**Questionnaire for subsequent telephone interview: English version.**

**The questions as below are about the headache associated with mobile phone use. Please answer the questions for your headache associated with a mobile phone use. (No.1~No.18)**

1. When did your headache associated with mobile phone use (HAMP) start?

_________ (years) (months) _______(months) (weeks) ago

2. When did your headache associated with mobile phone use develop? Please check all factors that bring on the headaches

(1) When mobile phone use was prolonged (Yes) (No)

(🡪 If you answered yes here answer No. 3 and skip No.4.)

(2) When mobile phone use was frequent (Yes) (No) (🡪If you answered yes here skip No. 3 and answer No.4.)

3. How long did it take to develop a headache associated with mobile phone use after starting a call?

(hour) (min) (e.g, 1hour)

4. How often did you use your mobile phone when headache associated with mobile phone use developed?

(daily) (hourly) times (e.g, hourly 3 times)

5. When did your headache associated with mobile phone use develop?

Headache developed during mobile phone calling. (Yes)/(No)

Headache developed after mobile phone calling. (Yes)/(No)

6. How often did headache associated with mobile phone use appear during or after your mobile phone calling?

(1/10 or less), (1/4 or less), (1/4 to 1/2), (1/2 to 3/4), (3/4or more), (always)

of all the mobile phone call

7. Where was the headache associated with mobile phone use located?

(1) Ipsilateral side when mobile phone was held to the ear

(2) Contralateral side when mobile phone was held to the ear

(3) Across the head

(4) No particular location but all over the area (migrating)

(5) Unilateral either way

(6) Irregular (inconsistent)

8. What was the headache associated with mobile phone use like? (Please check one statement that most accurately describe your headache associated with mobile phone use)

⬜ Pulsating and throbbing

⬜ Heavy and stiff

⬜ Tightening feeling like tying a band around your head

⬜ Sharp like pinpricking

⬜ Sudden and severe like hitting your head with a hammer

⬜ Creepy or crawling sensation

⬜ Other description

(If you answer “Other description” to questions, please describe the nature of your headache succinctly)

9 Did a burning sensation occur during or after your headache associated with mobile phone use? (Yes) (No)

(If yes, go to question No. 10. If no, skip question No. 10)

10. How often did a burning sensation occur during or after your headache associated with mobile phone use?

1. (1/10 or less), (1/4 or less), (1/4 to 1/2), (1/2 to 3/4), (3/4 or more), (always) of all mobile phone call
2. (1/10 or less), (1/4 or less) (1/4 to 1/2) (1/2 to 3/4) (3/4 or more) (always)

of all headache associated with mobile phone use

11. Did you experience dizziness when you had a headache associated mobile phone use?

(Yes) (No)

12. How bad was your headache associated with mobile phone use?

(1) Headache did not disturb usual daily activities (mild).

(2) Headache often disturbed usual daily activities, but I could perform more than half of my daily activities (moderate).

(3) I can’t perform my usual daily activities when I suffer these headaches (severe).

13. The headache associated with mobile phone use worsened by activities such as walking or climbing stairs? (Yes) (No)

14. Did you feel nauseated or sick to your stomach during your headache associated with mobile phone use? (Yes) (No)

15. Was your headache associated with mobile phone use more painful when you are in noisy surroundings? (Yes) (No)

16. Did light bother you a lot more than when you don’t have headache associated with mobile phone use?

(Yes) (No)

17. Did you smell differently during your headache associated with mobile phone use?

(Yes) (No)

18. Did you experience orbital or periorbital pains when you had headache associated with mobile phone use?

(Yes) (No)
